# P-112. Characterization of Herpes Zoster Cases and Sustained High Vaccine Efficacy Against Herpes Zoster Complications in Individuals Vaccinated with Recombinant Zoster Vaccine During A Long-Term Follow-Up Study

**DOI:** 10.1093/ofid/ofae631.319

**Published:** 2025-01-29

**Authors:** Rafael Leon, Desmond Curran, Ana Strezova, Javier Díez-Domingo, Sean Matthews, Manyee Tsang, Meng Shi, Agnes Mwakingwe-Omar

**Affiliations:** GSK, Wavre, Brabant Wallon, Belgium; GSK, Wavre, Brabant Wallon, Belgium; GSK, Wavre, Brabant Wallon, Belgium; FISABIO, Valencia, Comunidad Valenciana, Spain; GSK, Wavre, Brabant Wallon, Belgium; GSK, Wavre, Brabant Wallon, Belgium; GSK, Wavre, Brabant Wallon, Belgium; GSK, Rockville, MD, USA, Rockville, Maryland

## Abstract

**Background:**

ZOE-LTFU was an extension of two pivotal efficacy trials (ZOE-50/70) following participants vaccinated with adjuvanted recombinant zoster vaccine (RZV). Vaccine efficacy (VE) against herpes zoster (HZ) from 1 month post dose 2 up to 11 years was 87.7%. Here, we report characteristics of confirmed HZ cases during ZOE-LTFU, focusing on pain and complications.

**Figure:**
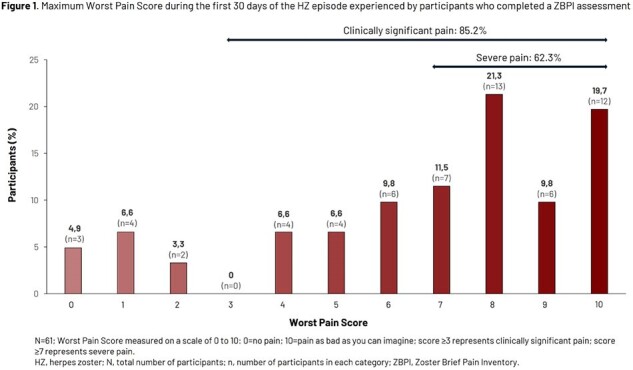

**Methods:**

ZOE-LTFU was a phase 3b, open-label study conducted in 18 countries between 2016 and 2023 (NCT02723773). Participants from the original randomized trials (NCT01165177 and NCT01165229) who received ≥1 dose of RZV were eligible. Pain experienced during an HZ episode was evaluated by the Zoster Brief Pain Inventory (ZBPI) until 90 days after first HZ visit or a 4-week pain-free period was achieved.

**Figure:**
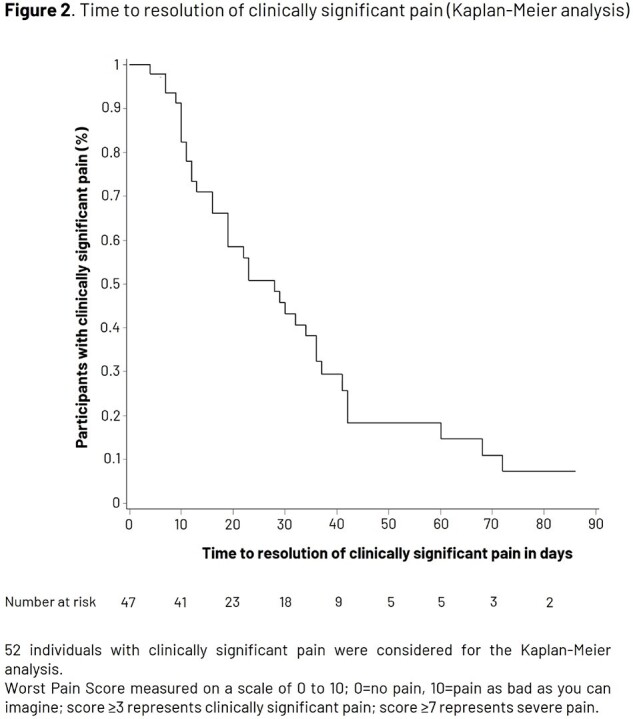

**Results:**

Of 7273 participants included in the primary cohort for VE analysis, 69 experienced a confirmed HZ episode and 61 had a first ZBPI evaluation within 16 days of rash start. Median age at first vaccination was 71.0 (50.0–87.0) years (N=69). Most participants (58/61, 95.1%) reported pain, while 52 (85.2%) and 38 (62.3%) reported clinically significant pain (score ≥ 3) and severe pain (score ≥ 7), respectively (Fig. 1). Mean Worst Pain Score was 6.7 (standard deviation 3.0). Clinically significant pain resolved after a median of 19 days; Kaplan-Meier analysis is shown in Fig. 2. Most participants (85.2%) received pain medication with a trend for longer duration of use with increasing age (Table 1). VE was 87.5% (95% confidence interval [CI] 64.8, 96.8) against post-herpetic neuralgia (PHN) and 91.7% (95% CI 43.7, 99.8) against non-PHN complications in participants ≥ 50 years; complications occurred in participants ≥ 76 years 6–10 years after vaccination (Table 2).

**Figure:**
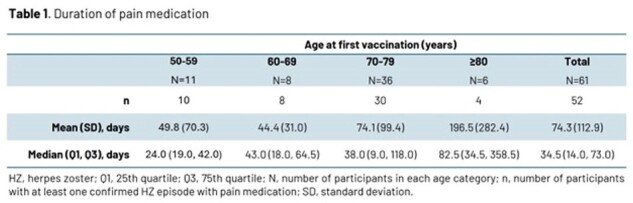

**Conclusion:**

The limited numbers of HZ cases in ZOE-LTFU were associated with less frequent HZ complications. Cases and complications were more common with older age. VE against PHN and non-PHN complications was sustained over 11 years of follow-up and comparable to the original studies. Overall, these findings support the long-term clinical benefit of RZV in prevention of HZ and its complications.

FUNDING: GSK

**Figure:**
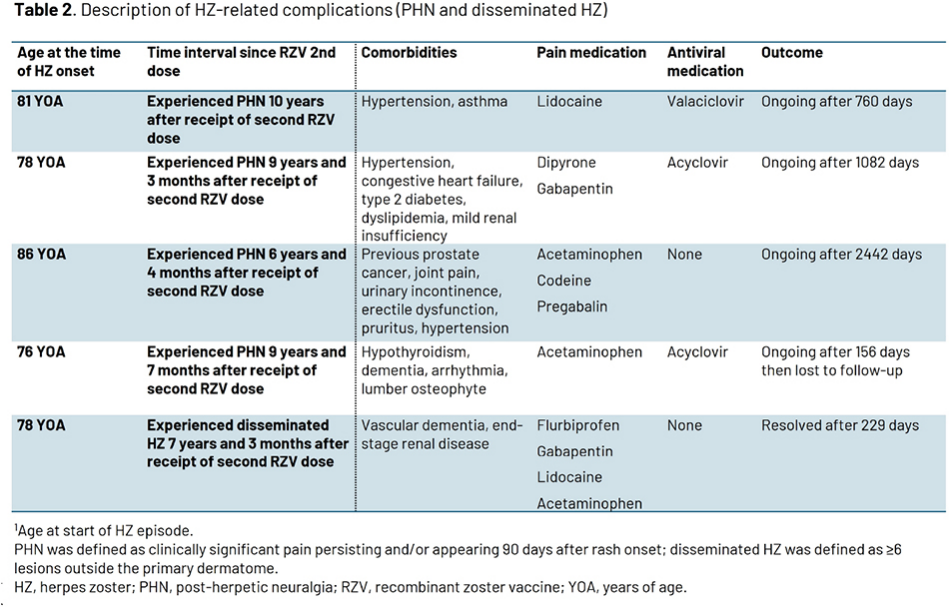

**Disclosures:**

**Rafael Leon**, GSK: Employee|GSK: Stocks/Bonds (Private Company) **Desmond Curran, PhD**, GSK: employee|GSK: Stocks/Bonds (Public Company) **Ana Strezova, MD, MSc**, GSK: Employee|GSK: Stocks/Bonds (Private Company) **Javier Díez-Domingo, MD, PhD**, GSK: Advisor/Consultant|GSK: Grant/Research Support|GSK: Honoraria|Moderna: Advisor/Consultant|Moderna: Grant/Research Support|Moderna: Honoraria|MSD: Advisor/Consultant|MSD: Grant/Research Support|MSD: Honoraria|Pfizer: Advisor/Consultant|Pfizer: Honoraria|SANOFI: Advisor/Consultant|SANOFI: Grant/Research Support|SANOFI: Honoraria **Sean Matthews, MSc**, GSK: Advisor/Consultant **Manyee Tsang, n/a**, GSK: Employee|GSK: Stocks/Bonds (Public Company) **Meng Shi, MS**, GSK: Employee **Agnes Mwakingwe-Omar, MD, PhD**, GSK: Employed|GSK: Stocks/Bonds (Private Company)

